# The Influence of Neck Muscle Activation on Head and Neck Injuries of Occupants in Frontal Impacts

**DOI:** 10.1155/2018/7279302

**Published:** 2018-05-09

**Authors:** Fan Li, Ronggui Lu, Wei Hu, Honggeng Li, Shiping Hu, Jiangzhong Hu, Haibin Wang, He Xie

**Affiliations:** ^1^State Key Laboratory of Advanced Design Manufacturing for Vehicle Body, Hunan University, Changsha 410082, China; ^2^Xiangya Hospital of Central South University, Xiangya Hospital, Changsha 410008, China; ^3^CRRC Zhuzhou Institute Co. Ltd., Zhuzhou, China

## Abstract

The aim of the present paper was to study the influence of neck muscle activation on head and neck injuries of vehicle occupants in frontal impacts. A mixed dummy-human finite element model was developed to simulate a frontal impact. The head-neck part of a Hybrid III dummy model was replaced by a well-validated head-neck FE model with passive and active muscle characteristics. The mixed dummy-human FE model was validated by 15 G frontal volunteer tests conducted in the Naval Biodynamics Laboratory. The effects of neck muscle activation on the head dynamic responses and neck injuries of occupants in three frontal impact intensities, low speed (10 km/h), medium speed (30 km/h), and high speed (50 km/h), were studied. The results showed that the mixed dummy-human FE model has good biofidelity. The activation of neck muscles can not only lower the head resultant acceleration under different impact intensities and the head angular acceleration in medium- and high-speed impacts, thereby reducing the risks of head injury, but also protect the neck from injury in low-speed impacts.

## 1. Introduction

Vehicle accidents kill approximately 1.25 million people each year, and another 20 to 50 million people suffer nonfatal injuries, with the costs accounting for 1–3% of the gross domestic product of most countries [1]. Head and neck injuries are the most severe injuries in vehicle accidents [2, 3]. The study by NHTSA (National Highway Traffic Safety Administration) [4] found that nearly 15,000 passenger vehicle occupant deaths occur annually in the United States due to car accidents involving frontal crashes.

With the development of passive safety techniques, occupants are better protected during vehicle accidents. To date, however, the muscle activation behavior of occupants has not been considered in crash tests. Muscle activation plays an important role in human body function and will generate forces that affect the occupants' dynamic response upon colliding with an airbag [5, 6] and their biomechanical responses [7, 8], especially in a low-speed impact, which will increase the cervical stretch tolerance and vary the injury locations from the lower cervical spine to near the head [9]. Additionally, the stress on the upper cervical ligament in a frontal impact would be significantly reduced by the force of muscle contraction [10]. However, the injury mechanism influenced by the muscle contracting force remains unclear, especially for the injury of soft tissues in the neck. Recent studies on head and neck injuries have mostly focused on a rear-end impact, which would slow the progress of neck injury prevention. Although the observed frequency of neck injuries in a rear-end impact versus a frontal impact of comparable severity was higher [11], the effect of the muscle activation force should not be ignored for a low-speed frontal impact [12]. In a severe frontal impact, the neck will acquire an S shape, as observed in rear-end impacts, leading to unclear injuries to neck tissues [13]. Thus, we need to study the muscle activation in head and neck injuries of occupants in frontal impacts.

Previous methods for studying the influence of muscle activation on neck injuries have many shortcomings: the mechanical dummies have lower biofidelity; PHMS (post mortem human subjects) tests cannot represent muscle activation behavior, even though they can be used to simulate a high-speed impact; and volunteer tests can only be carried out in a very low-speed impact for ethical reasons, although muscle activation can still be observed. Consequently, FE model simulation seems to be able to overcome the deficiencies of the former methods: it can easily obtain important information, including dynamic parameters and even the stress and strain at different magnitudes and impact speeds.

Over the past several decades, many neck FE models have been developed. A human head-neck model was developed with the bony vertebrae modeled by shell elements, and the relevant muscles and ligaments were modeled by membranes and spring-damper elements, respectively [14]. Since solid muscles can affect the stabilization of the body due to compression stiffness and inertial effects, which significantly lessens the need for muscle activation in an impact, three-dimensional solid muscle models with continuous material properties in which the friction among muscles is precisely realized have been adopted. Ejima et al. [5] simulated the passive solid cervical muscle tissues without considering anisotropy, that is, the nonviscoelastic properties of muscles. Frechede et al. [6, 15] considered the characteristic anisotropy of passive muscle tissues but defined the mechanical properties as linear elasticity. Hedenstierna [16], in Sweden, implemented muscle activation realized by a Hill element into passive solid neck muscles simulated by nonlinear elasticity and viscoelastic properties. Famous full-scale human FE models, such as the THUMS (total human model for safety) [17, 18] and the GHBMC (Global Human Body Models Consortium mid-sized male full-body model) [19, 20], included both active and passive muscle properties. However, the FE models developed during the early stages were not sufficiently accurate because of structural shortcomings and a lack of muscle activation [5, 6, 14, 15]. A model developed by Hedenstierna [16] included no other parts of the human body, and the biofidelity of the transition between C7 and the thorax was not good enough. The THUMS and GHBMC models suffer from low computational efficiency and the geometric shortcomings of neck muscles [17–20]. Thus, to study the influence of neck muscle activation on neck injuries, a model overcoming the mentioned disadvantages is desired.

The aim of the present paper was at studying the influence of neck muscle activation on head and neck injuries of vehicle occupants in frontal impacts by using a mixed dummy-human FE model. When developing the model, the head-neck portion of the Hybrid III dummy model was replaced by a well-validated head-neck FE model with passive and active muscle characteristics. The mixed dummy-human FE model was validated by the NBDL (Naval Biodynamics Laboratory) in 15 G frontal volunteer tests. The mechanism by which neck muscle activation affects the head and neck injuries of vehicle occupants in frontal impacts of three major intensities, low speed (10 km/h), medium speed (30 km/h), and high speed (50 km/h), was studied.

## 2. Methods and Materials

A mixed dummy-human FE model was developed by replacing the head-neck portion of the Hybrid III model with a well-validated head-neck human model that can simulate the activation behavior of neck muscles. The mixed dummy-human FE model was then validated via frontal impact simulation in NBDL experiments [21, 22]. To study the mechanism by which muscle activation affects head and neck injury, the mixed dummy model was used to examine three major frontal impact intensities (i.e., 10 km/h, 30 km/h, and 50 km/h).

### 2.1. Development of the Mixed Dummy-Human FE Model

#### 2.1.1. Head-Neck Human FE model

**Figure 1:**
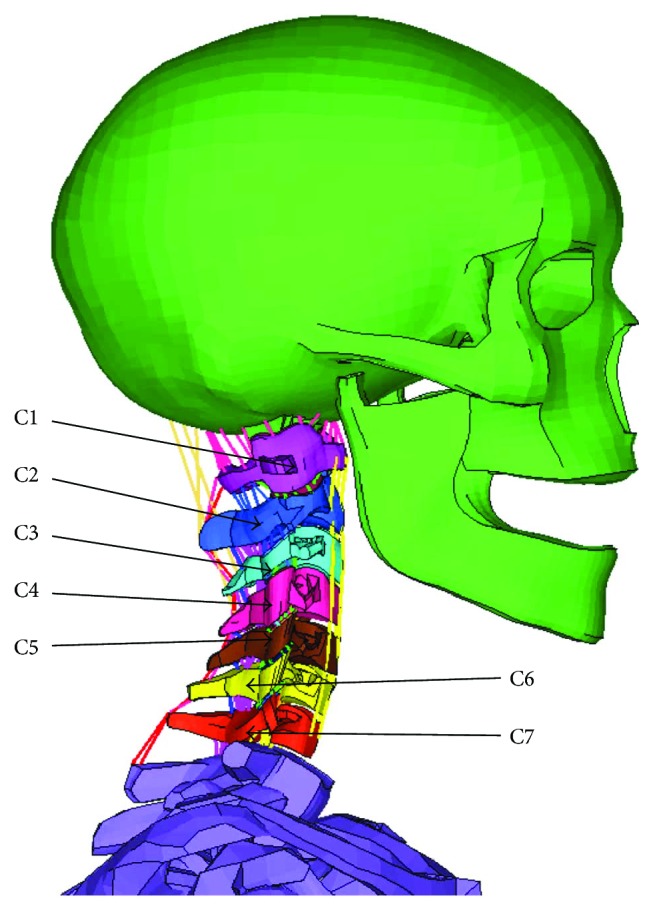
Basic head-neck human FE model.

**Figure 2:**
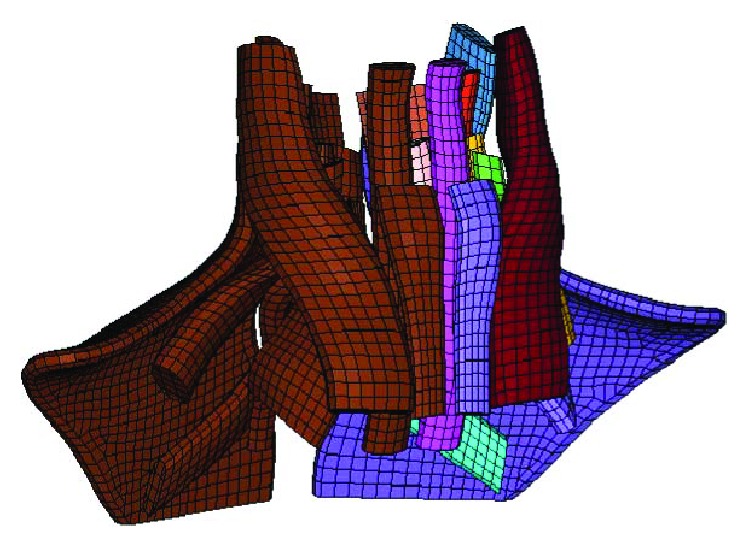
Neck muscle model.

The head-neck human FE model represents a 50th percentile male, and it was developed from the basic head-neck model (Figure 1) validated by Yang and Yao [23]. This basic model has a detailed anatomical structure, including a skull, cervical vertebra (C1–C7), intervertebral discs, facet joints, cervical ligaments, and muscles that were modeled by 1D beam elements. The skull and shoulder were defined to be rigid because this model is mainly used for neck injury-related studies. The detailed solid neck muscle model (Figure 2) was developed based on neck MRI images of a 50th percentile adult male [24] and integrated into the basic head-neck human FE model using a kriging method by mapping the origins and terminations as well as coordinate information of the neck muscles of volunteers to the basic head-neck model (the original model includes detailed thorax geometry) [25, 26]. Kriging is a type of optimal interpolation first proposed by DG Krige, a geologist in South Africa. For detailed information about the kriging method, please refer to the literature [27].

**Figure 3:**
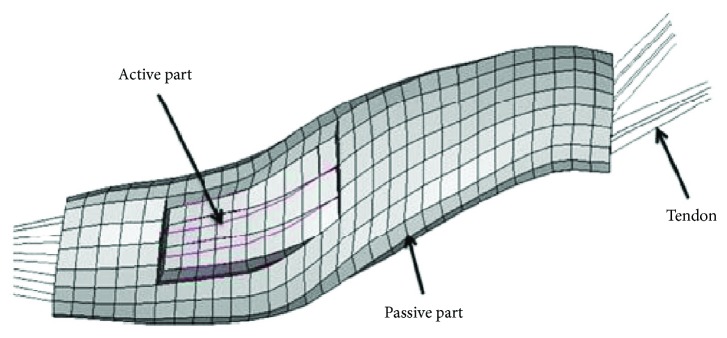
A coupled single muscle.

**Figure 4:**
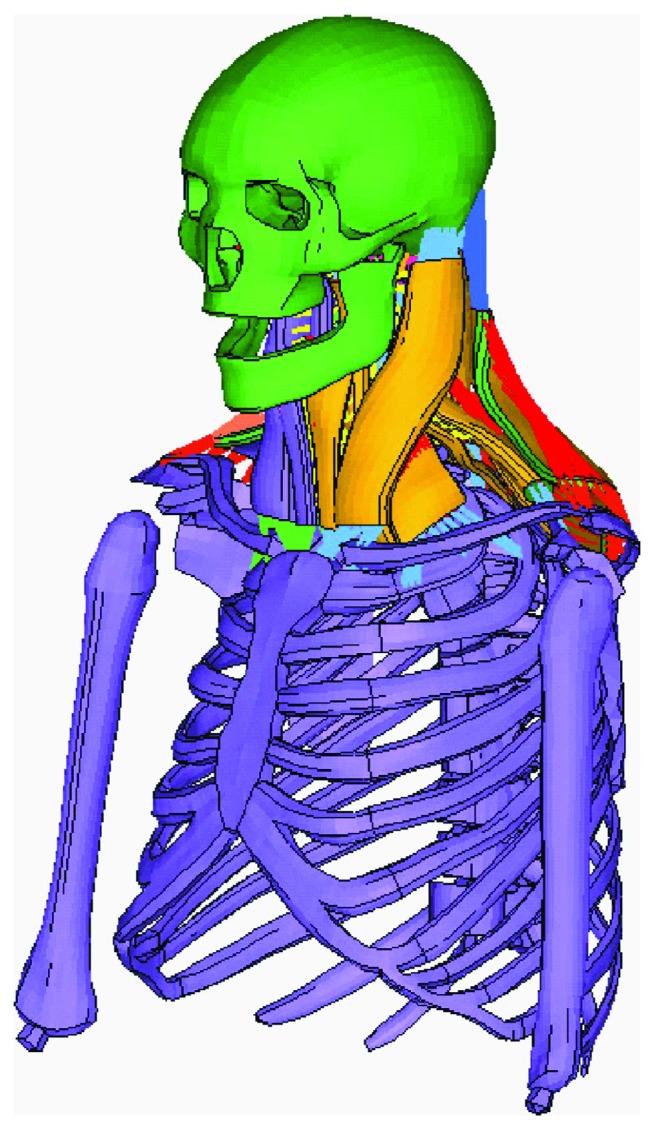
Head-neck human FE model.

A single-muscle FE model consisted of three parts: the tendon modeled by a beam element, the passive muscle belly modeled by a solid element, and the active muscle modeled by a beam element (Figure 3). The active part of the model was merged into the passive part with shared nodes. The active part was modeled by a Hill-type element defined as MAT_156 material in the LS-DYNA software, and the passive part was defined as a hyperelastic model in Ogden material (MAT 77 in LS-DYNA). A detailed material definition can be found in a study conducted by Li et al. [25].  Figure 4 shows the head-neck human FE model developed from models illustrated above.

#### 2.1.2. Hybrid III Dummy FE Model

**Figure 5:**
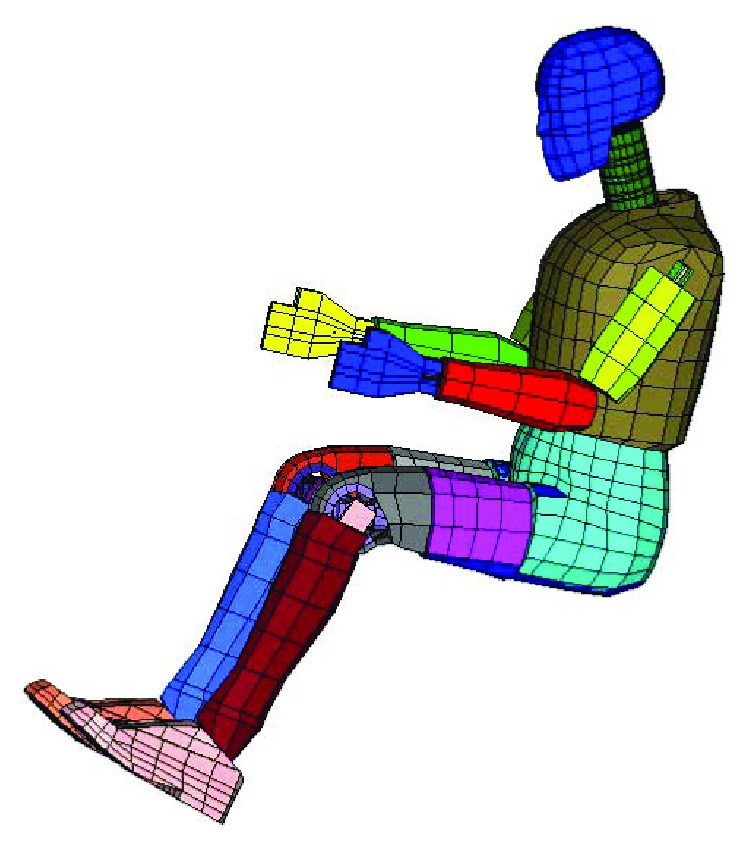
Hybrid III dummy.

The Hybrid III mechanical dummy was developed by General Motors in 1976 and was widely used by vehicle companies. The relative FE model applied in the present study was a commercial model developed using the LS-DYNA software. The model includes a head, neck, chest, abdomen, pelvis, and limbs, consisting of 7784 nodes and 4412 elements (Figure 5).

### 2.2. Mixed Dummy-Human FE Model

**Figure 6:**
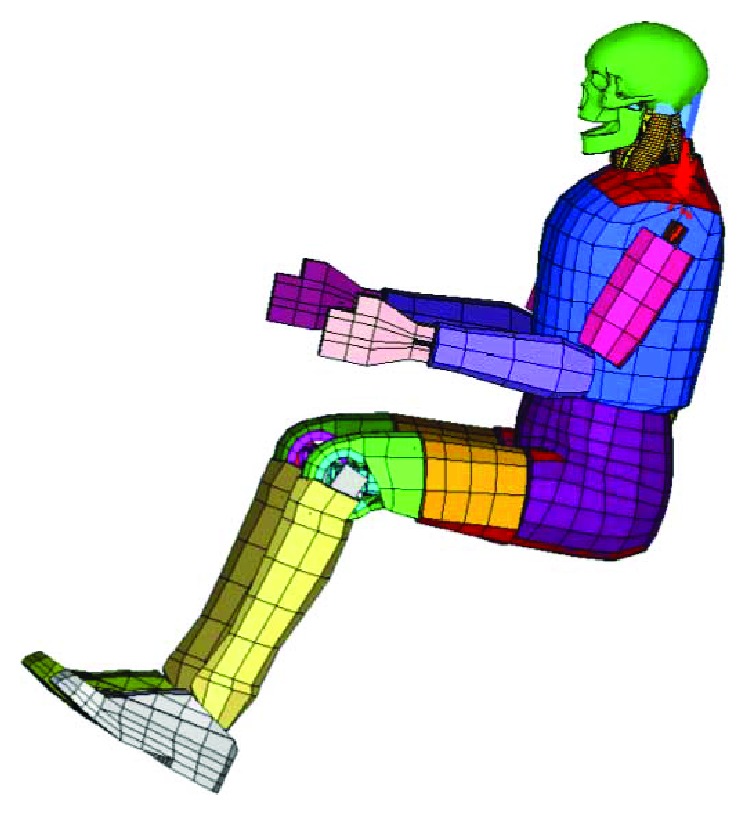
The mixed dummy-human FE model.

In terms of the computational efficiency, a full-scale human FE model such as GHBMC or THUMS, with detailed soft tissues, such as muscles, brains, and the haslet, is time consuming. Although the mechanical dummy FE model has good computational efficiency, it has less biofidelity than does the full-scale human FE model. In this study, we decided to combine the advantages of both models by developing a mixed dummy-human FE model, replacing the head-neck portions of Hybrid III with the head-neck of a human FE model, as previously mentioned. This mixed model has good computational efficiency and good biofidelity for studying the effects of muscle activation on head and neck injuries and other related biomechanical studies. The kriging method was also used to develop the complex model. The outlines and coordinate information of the T1 of the head-neck human FE model were mapped to the T1 of the dummy parts and connected with anatomical joints [27, 28]. The rigid thorax and T1 of the head-neck human FE model and the dummy FE model were used to form a corresponding coordinate system for adjusting the posture of the mixed FE model. The mixed dummy-human model has a precrash sitting posture similar to that of the Hybrid III FE dummy, as shown in Figure 6.

### 2.3. Validation of the Mixed Dummy-Human FE Model

#### 2.3.1. NBDL Experiment

**Figure 7:**
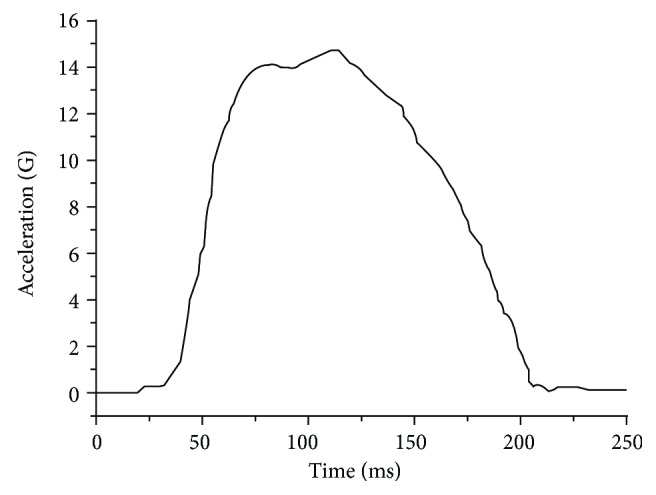
Loading acceleration in NBDL tests.

The mixed dummy-human FE model was validated based on 15 G NBDL frontal volunteer tests. Volunteers (young, well-trained marines) were seated in an upright position on a rigid seat mounted on a HYGE accelerator and exposed to a short-duration acceleration that simulated a frontal collision. Accelerometers and photographic targets were mounted on the subject and used to monitor the resultant three-dimensional motions of the head and T1. A detailed description of the instrumentation and the test methods were provided by Ewing et al. [21, 22]. The peak value of the sled acceleration (i.e., the mean value of the sled acceleration-time history) was 15 G, and the speed change was greater than 17 m/s (Figure 7). The dynamic responses of the head and neck of the volunteers were recorded. The experimental corridors used in the validation process, obtained from the NBDL experiments [22], were expressed as the average volunteer response plus or minus the standard deviation [29].

#### 2.3.2. Simulation Setting

**Figure 8:**
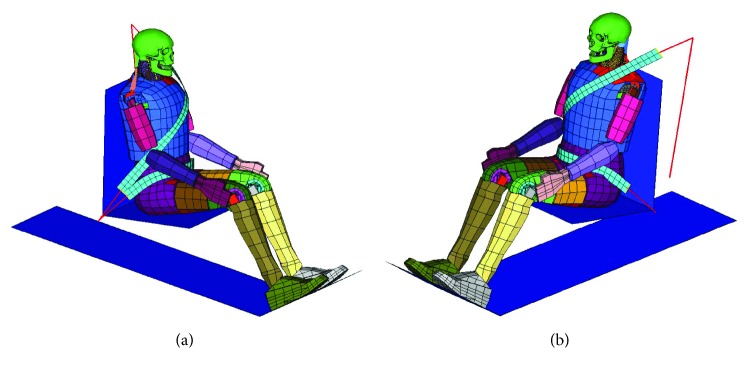
The mixed dummy-human FE model in an automotive posture.

**Figure 9:**
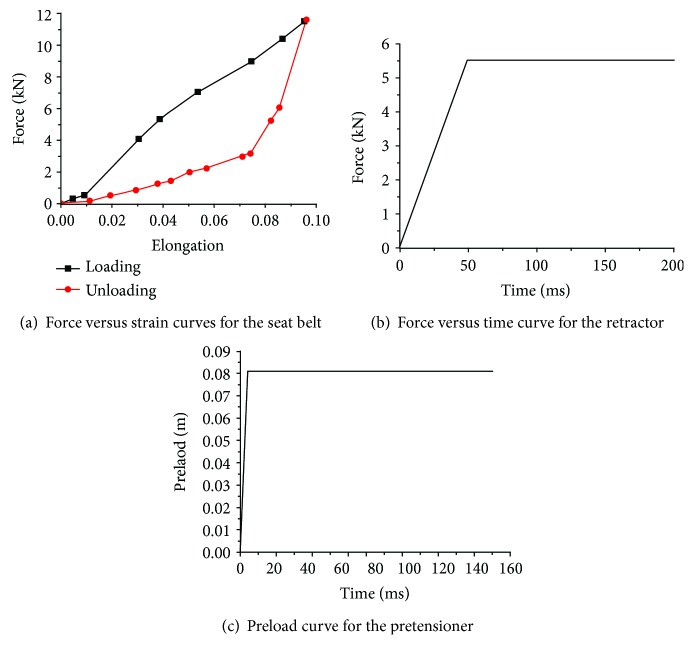
Loading curves applied to the seat belt system.

Since the seating posture has a great influence on the head injury of an occupant [30], to minimize its impact, the mixed dummy-human FE model was set in a normal automotive posture (Figure 8) in a gravity field, as in the NBDL experiment. The dummy was seated on a rigid seat that was connected to a rigid plate representing the vehicle. The lower arm was placed on the thigh. The configuration about the occupant restraint system was mainly adopted from the commercial FE vehicle model [31] used in this study. The seat belt included a retractor, slip rings, pretensioner, a ribbon modeled by 1D beam elements and 2D shell elements, and anchor nodes connected to rigid seats. The 1D beam elements were able to simulate the sliding effect of the slip rings. The force versus engineering strain curves for the seat belt loading and unloading, the force versus time curve for the retractor, and the preload curve for the pretensioner are described in Figure 9.

The active contracting forces that model muscle activation were activated by *A*(*t*). However, the time history of muscle activation was not immediately activated at the time of the collision. Instead it was

(1) $$\begin{array}{c} t_{act}=t_{trigger}+t_{reflex}, \end{array}$$

where *t*_trigger_ is defined as a certain sensory threshold time and *t*_reflex_ is a neural reflex time that describes the excitation and activation dynamics, respectively. The two constants throughout the study were set at 30 ms and 40 ms for *t*_trigger_ and *t*_reflex_, respectively, by methods referring to a study conducted by van der Horst [29].

**Figure 10:**
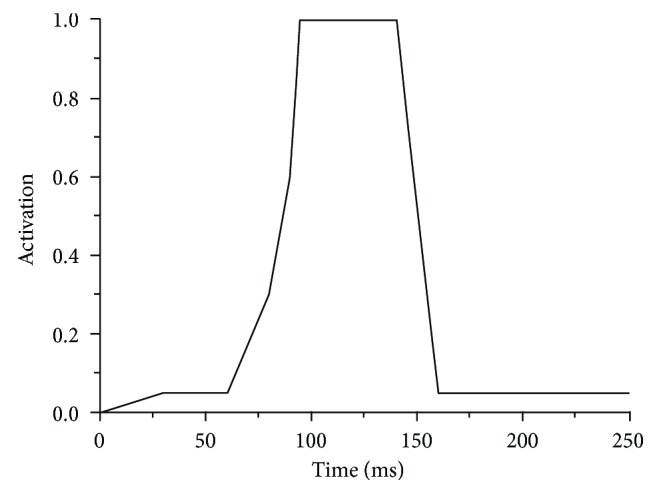
Muscle activation.

The *A*(*t*) time history, including parameters such as the maximum activation level (Act_max_ = 1), the time the first maximum activation level is reached (*t*_peak_ = 95 ms), and the time the end of activation is reached (*t*_end_ = 250 ms), are shown in Figure 10.

To make the dynamic responses of the head and neck clearly understood, the methods used to set the responses in the simulation are shown in Table 1.

The impact acceleration curve (Figure 7) generated from the hydraulic impactor was used as the input for the simulation, and the run time of the simulation was set to 200 ms because the dynamic indicators in the NBDL experiment recovered to a low level in approximately 200 ms. The time histories of the head rotational angle, neck rotational angle, head angular acceleration, and head resultant acceleration were compared with the experimental curves and the simulation results of head-neck model by Cao et al. [26].

### 2.4. Influence of Neck Muscle Activation of Head and Neck Injuries under Various Intensities

**Figure 11:**
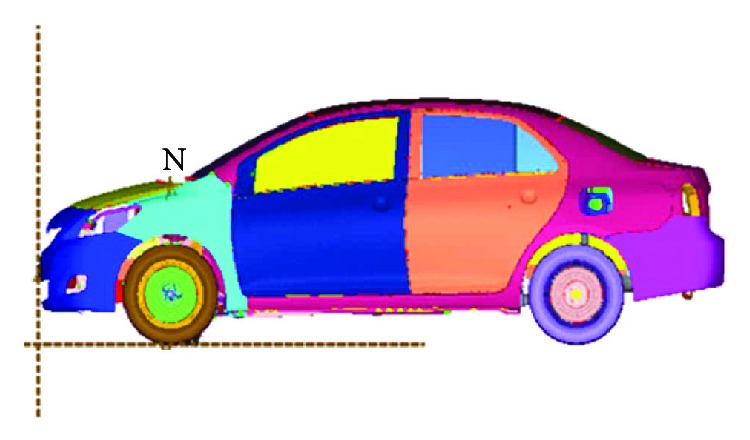
The validated vehicle model.

**Figure 12:**
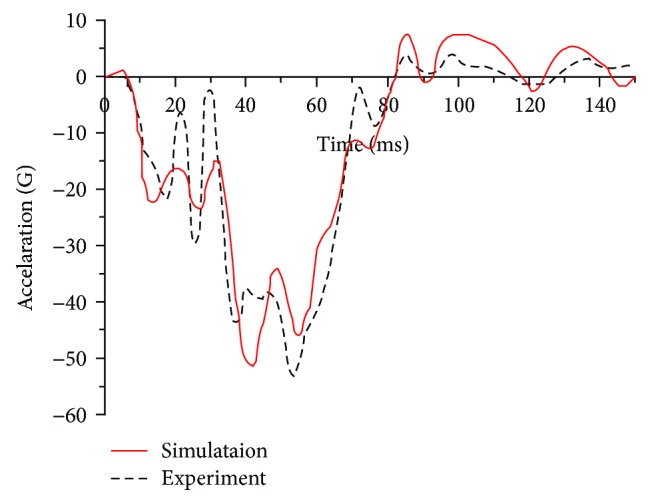
Vehicle acceleration curves.

**Figure 13:**
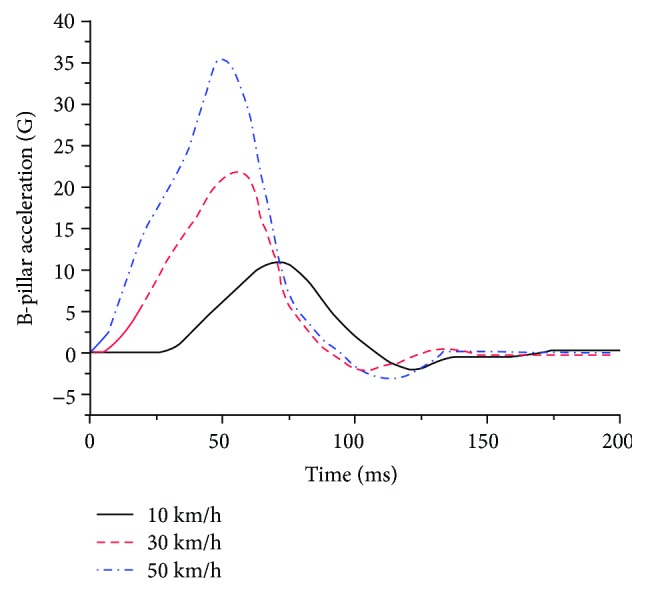
Loading acceleration curves for a frontal impact simulation.

The influence of neck muscle activation on head and neck injuries in a frontal crash was considered for three impact intensities: 10 km/h, 30 km/h, and 50 km/h, representing low-speed, medium-speed, and high-speed impact velocities in a 100% full vehicle frontal impact simulation, respectively. The effectiveness of the vehicle (Figure 11) was successfully validated by comparing the acceleration of the rear seat in a 100% full vehicle frontal impact simulation at 50 km/h to that in the experiment (Figure 12) [31]. The impact pulses of the B-pillar (Figure 13), retrieved from the simulations of a 100% full vehicle frontal impact at the three intensities mentioned above, were used as the input for the simulations.

The muscle activation was the same as that utilized to validate the mixed dummy human FE model, and the run time for the simulations was set to 200 ms.

## 3. Results

### 3.1. Model Validation

**Figure 14:**
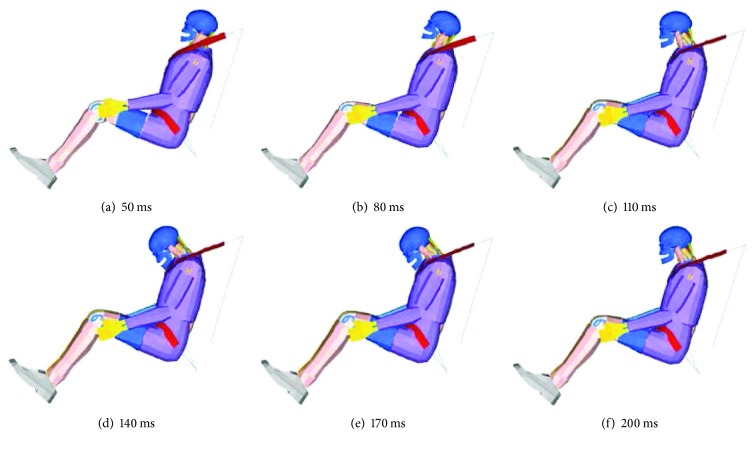
Dynamic responses in a frontal impact simulation.

The overall dynamic responses of the present model are shown in Figure 14. From 0 ms to 50 ms, the model was in a static equilibrium state because the impact pulse in this period was set to 0 for presimulation, with gravity alone acting on the mixed model (Figure 14(a)). After this period, as the impact pulse increased, the torso of the dummy moved forward and separated from the seat back (Figure 14(b)), while the head moved hysterically with a slight neck extension because the neck muscles were not ready to move. However, the muscle began to activate and the head moved together with the thorax and neck. Beginning at 105 ms, according to the behavior of the restraint system, the torso movement was limited while the head began to wrap forward, with the neck flexion assuming an S shape (Figure 14(c)). In this period, the neck muscles were fully activated but still could not provide adequate torque to maintain the stability of the head. The head then reached an extreme position at 165 ms (C shape in Figure 14(d)) and began to wrap backward as the neck extended. Between 181 ms and 200 ms, the head and neck gradually rebounded due to muscle traction.

**Figure 15:**
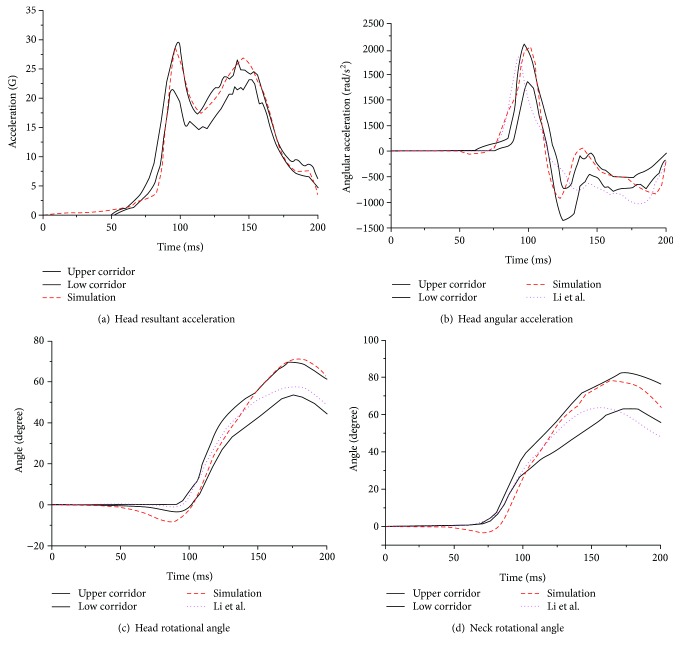
Dynamic response curves of the head and neck in a frontal impact simulation.

The dynamic responses of the head and neck were consistent with the NBDL experimental curves. The time points (approximately 98 ms and 150 ms) for the peak value of the resultant acceleration of the head in the volunteer response were accurate enough, but the acceleration value was only slightly above the curve during the initial period due to the precrash equilibrium (Figure 15(a)). The value of the first peak fell exactly in the channel and the second exceeded it by approximately 6%. Other outputs showed a similar tendency. The head angular acceleration was in good agreement with the curves apart from a small amount of overload at the end (Figure 15(b)). Compared to the output from the study conducted by Cao et al. [26], the amount outside the curve at the final stage was much smaller. From 50 to 100 ms, the head rotational angle (Figure 15(c)) was below the range of the channel and the time that the maximum value was reached was almost the same as that in the experimental results, while the head rotational angle in the study of Cao et al. fell exactly within the channel, and its peak value was 18.9% lower than that of the present study. The neck rotational angle (Figure 15(d)) was outside the curve between 50 ms and 100 ms but fell within the channel before reaching the peak value. Although the maximum value stayed almost the same as for the volunteer response, the time the maximum was reached was 10 ms early. In contrast to the neck rotational angle in the study of Cao et al., the trend of the neck rotational angle in the present study was more consistent with that of the NBDL experimental curves.

### 3.2. Muscle Activation Behavior

**Figure 16:**
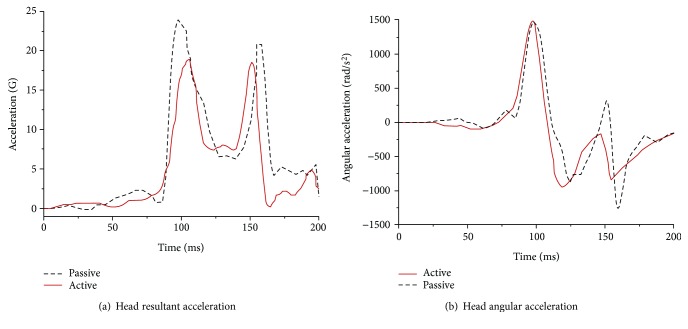
Dynamic response curves of the head and neck in a frontal impact simulation under 10 km/h.

**Figure 17:**
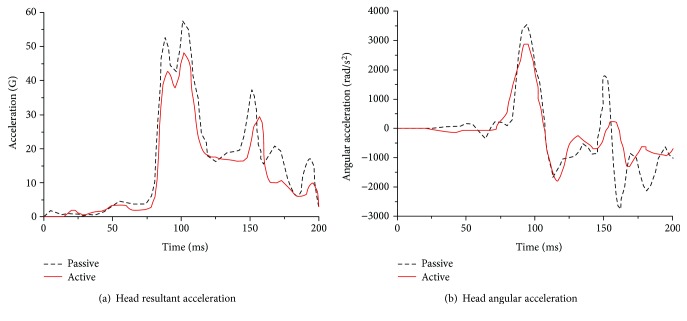
Dynamic response curves of the head and neck in a frontal impact simulation under 30 km/h.

**Figure 18:**
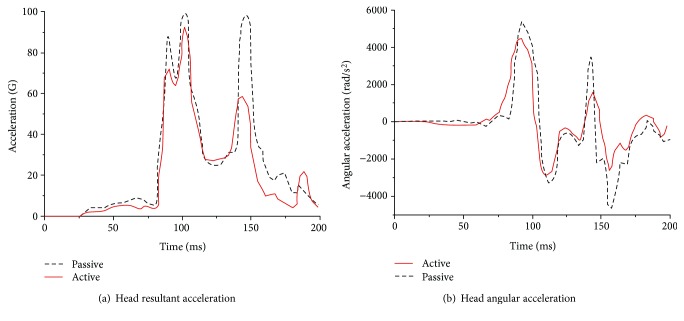
Dynamic response curves of the head and neck in a frontal impact simulation under 50 km/h.

A comparison of the head dynamic responses with the muscles activated or not activated (denoted the active or passive model, resp.) is shown in Figures 16–18. In a low-speed (10 km/h) frontal impact simulation, two peak values of the resultant acceleration were observed in both the active and passive models. The resultant acceleration reached its first peak of 18.9 G at 105 ms in the active model and at 24 G at 97 ms in the passive model. The second peak time in the passive model was 6 ms later than that in the active model, and the acceleration value differed by 2.5 G. The maximum angular acceleration reached 1496 rad/s^2^ at 98 ms in the active model, and it was 1478 rad/s^2^ at 103 ms in the passive model.

For the medium-speed (30 km/h) frontal impact simulation results, the peak head resultant accelerations in the active and positive models occurred at 89 ms and 97 ms, respectively; the first peak of the former was reduced by approximately 18%, and the second peak value was reduced by more than 27%. Meanwhile, the peak angular acceleration in both models was observed at the same time. However, the peak angular acceleration in the passive model was approximately 20.8% higher than that in the active model.

In a high-intensity impact (50 km/h), a dual peak was also found between 90 ms and 102 ms in both models for the outputs of head resultant accelerations, but the peak in the passive model was approximately 24% higher than that in the active model. During the extension period, the peak in the passive model was 97.8 G, while it was 57 G in the active model, which was much lower. The peak angular acceleration in both models occurred at approximately 92 ms, while the peak in the passive model was 15.3% higher than that in the active model.

**Figure 19:**
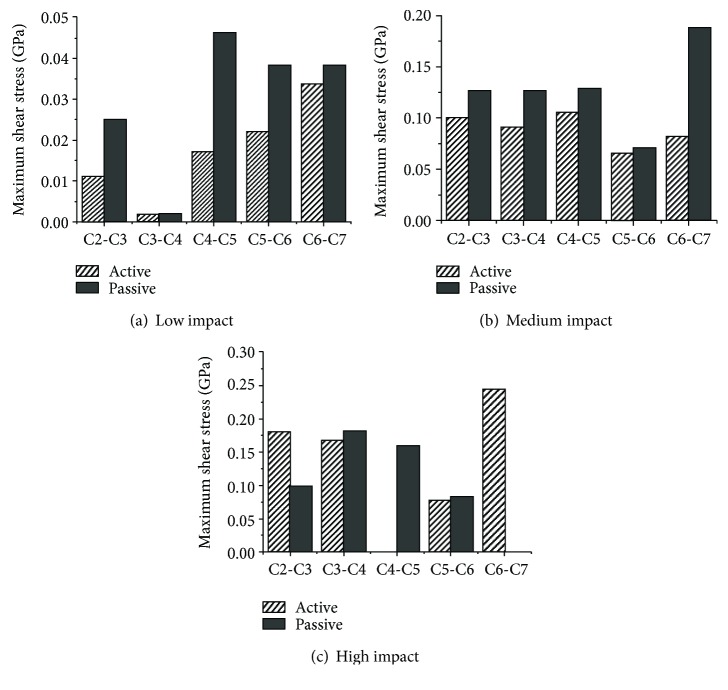
Maximum shear stress of intervertebral discs at various impact intensities.

**Figure 20:**
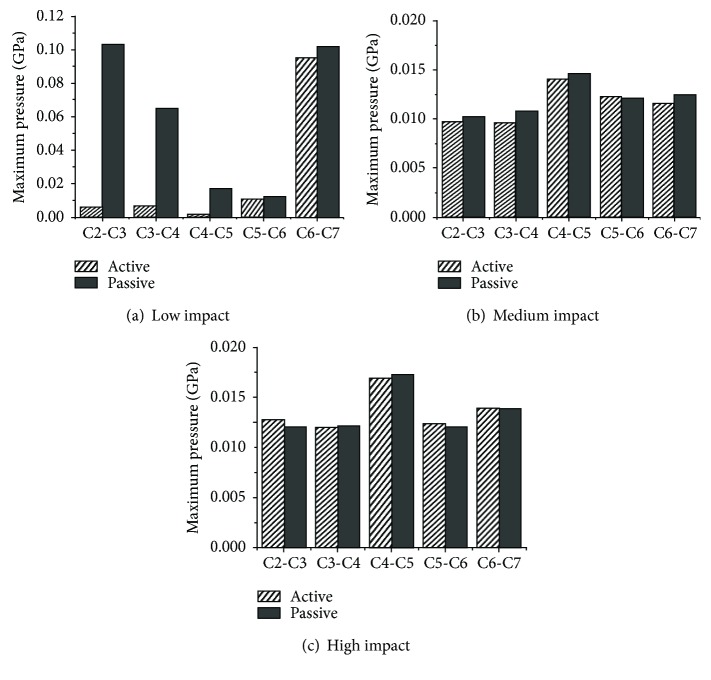
Maximum pressure of intervertebral discs at various impact intensities.

**Figure 21:**
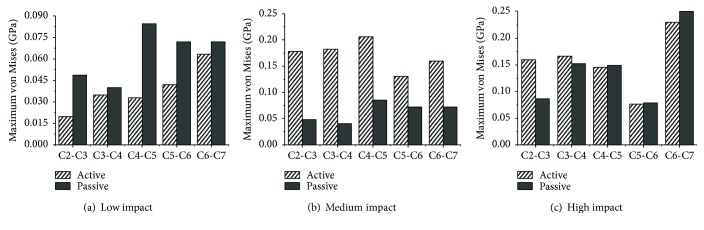
Maximum von Mises stress of intervertebral discs at various impact intensities.

The stresses on the intervertebral discs are shown in Figures 19–21. Overall, as the impact intensity increased, the stress on the intervertebral discs increased. The stress in the active model was much lower compared to that in the passive model especially for the C2-C3, C3-C4, and C4-C5 discs in a low-speed impact intensity crash. For the C2-C3 disc of the model in a low-speed impact, the maximum pressure was 0.103 GPa in the passive model, more than 17 times the 0.005684 GPa observed in the active model. The maximum shear stress in the passive model was 0.0252 GPa, exceeding by 129% the 0.011 GPa in the active model. Additionally, the maximum von Mises stress of the C4-C5 disc in the passive model was approximately 1.58 times higher than that in the active model. However, the maximum shear stress on the C2-C3 and C4-C5 discs, the maximum pressure in the C2-C3 and C5-C6 discs, and the maximum von Mises stress in the C2-C3 and C3-C4 discs in high-speed impact showed the opposite tendency. For the C2-C3 disc, the maximum von Mises stress in the active model was approximately 33% higher than that in the passive model. The maximum shear stress and the maximum pressure in the active model in a medium-speed impact were lower than those in the passive model, while the maximum von Mises stress showed the opposite tendency.

## 4. Discussion

In the present paper, a mixed dummy-human FE model was developed. This model was validated according to NBDL volunteer frontal impact experiments. The influence of neck muscle activation on head-neck injuries of occupants in frontal impacts was analyzed using the mixed dummy-human FE model in three impact intensities, that is, 10 km/h, 30 km/h, and 50 km/h. The dynamic responses of head and biomechanical responses of cervical intervertebral discs, including maximum von Mises stress, maximum shear stress, and maximum pressure, were compared in terms of whether the neck muscle was activated.

The mixed dummy-human FE model turned out to have good biofidelity for the simulation compared to the NBDL experimental corridors. The mixed model showed good computational efficiency for a 200 ms frontal impact simulation, requiring approximately 5 hours using a computer CPU with a 40-core Intel(R) E5-2670 v2 at 2.50 GHz processor. Because of the dummy torso behavior, the head-neck dynamic responses were more reliable, as the previous human head-neck single model could only be loaded on C7 and the driving behavior between vertebras or between torso and neck could thus not be simulated. In the study conducted by Li et al. [26, 28, 32], using only the previous human head-neck model, two peaks for the head resultant acceleration were observed instead of one (as in the present study), and this phenomenon was also seen in similar studies [8] when the simulation used a head-neck model without torso parts.

In terms of the head dynamic responses for the different frontal impact intensities, including the linear acceleration and angular acceleration that were greatly relevant to the head HIC [33–35] and head AIS injury level [36], respectively, activation of neck muscles during a frontal impact seems to be an important factor in reducing the head injury risk. The peak head resultant acceleration in the passive model was 27% (low intensity) to 24% (high intensity) greater than that in the active model, which suggested that muscle activation plays a more important role in reducing the HIC of the head in a low-intensity impact. According to a study by Ommaya [36], the head angular acceleration has a definite relationship with the AIS level, as illustrated in Table 2. In our study, muscle activation seemed to be more effective in reducing the head AIS level in medium- and high-intensity impacts. Neck muscle activation reduced AIS from level 3 in the passive model to level 2 in the active model and from level 5 in the passive model to level 4 in the active model in medium- and high-speed impacts, respectively. However, in a low-speed impact, humans have a low risk of head injury, and muscle activation has few effects. Consequently, we could conclude that the activation of neck muscle could reduce head HIC, especially in a low-intensity frontal impact, but would have slightly less effect in medium- and high-intensity impacts. The neck activation system may reduce the head AIS level, especially in medium- and high-intensity impacts. Considering the design of the restraint system, although the explosion time of an airbag is designed according to a test dummy without neck muscle activation, it can still efficiently protect actual human occupants from serious head injuries.

Studies have suggested that an injury of the intervertebral disc could occur due to local shear, compression, or tension forces caused by movement of the vertebral bodies [11, 37]. In the present study, neck muscle activation showed an obvious effect in reducing intervertebral disc shear stress at almost all impact intensities, and in reducing the pressure stress at low and medium impact intensities. To date, however, there are insufficient studies to illustrate the relationship between the reduced stresses or pressures and AIS level of head injuries. The main function of the neck muscle system is to maintain the stability of the head and prevent the neck from excessive shear and pressure loads. In a low-speed impact, when a muscle activates, one component of the force is able to resist the shear load, and the other successfully reduces the axial pressure since the extension and flexion of the neck is not severe. When the impact intensity increases, the dynamic indicators become larger and the muscles must provide more force to maintain the stability of the head and prevent disc injuries due to shear stress and axial pressure. However, as the impact intensity increases, the pressure reduction due to neck activation varies from 94.4% to 11.2%, and the maximum shear stress varies from 63.06% to 56%, which suggests that neck activation has less protective effect. Meanwhile, it is worth noting that muscle activation in a high-intensity impact increases the stress on the upper intervertebral disc between C2 and C3. The reasons may be that the muscle activation would become larger because the severe inflexion occurring in the neck will increase with the impact intensity. Once the active force exceeds a certain value and surpasses the loads from the vertebral bodies, the intervertebral shear stress and pressure would increase concomitantly. This phenomenon obviously occurred in the upper neck in a high-speed frontal impact. The additional axial compression can reduce the shear stiffness of the cervical disc and make it easier for the shear-type soft tissue injuries to occur [29]. From the present study, we can conclude that the muscle activation behavior can prevent serious neck injuries in a low-speed frontal impact but that the risk of injury in the upper neck may be increased in a high-speed frontal impact. We are not sure whether the muscle activation behavior can raise or reduce the lower neck injuries in a high-speed frontal impact, considering the uncertain trends of shear stress and pressure between the active and passive models.

In this study, because T1 is defined as rigid according to the Hybrid III dummy characteristics, the connection between C1 and T1 seems to have less biofidelity in terms of the force and torque transition between the rigid and flexible parts. Another limitation of the present study is that the high impact intensity was set to 50 km/h but a higher impact speed was not studied. This is due to the poor computational stability of the soft tissue in simulations at higher intensities.

## 5. Conclusion

The mixed dummy model has good computational efficiency and biofidelity for studying the effects of muscle activation on related head and neck injuries.

The activation of neck muscles can lower the head resultant acceleration under different impact intensities and the head angular acceleration in medium- and high-speed impacts, thereby reducing the risks of head injury.

The activation of neck muscles can also protect the neck from shear and compression injuries in a low-speed fontal impact. As the impact intensity increases, the protective effect of muscle activation on head and neck injuries is decreased. Although neck muscle activation can prevent shear stress and pressure in a medium-speed frontal impact, it may fail to prevent other types of injuries. In a high-speed frontal impact, neck muscle activation may even contribute to the risk of shear and compression injury of the upper neck.

## Figures and Tables

**Table 1:** Response settings.

Response parameters	With respect to
Head resultant acceleration^*a*^	Global system
Head angular acceleration^*b*^	System in T1
Head rotational angle^*c*^	System in T1
Neck rotational angle^*d*^	System in T1

^a^Resultant acceleration of the center of gravity of the head (CG). ^b^Angular acceleration along a straight line connecting the occipital condyles (OC) to CG in the sagittal plane. ^c^Angle of a straight line connecting OC to CG in the sagittal plane. ^d^Angle of a straight line connecting OC to T1 in the sagittal plane.

**Table 2:** Relationship between AIS level and injury tolerance of head angular acceleration [36].

AIS level	Injury tolerance
AIS1	$\ddot{a}$ < 1700 rad/s^2^
AIS2	$1700\;rad/s^{2}<\ddot{a}<3000\;rad/s^{2}$
AIS3	$3000\;rad/s^{2}<\ddot{a}<3900\;rad/s^{2}$
AIS4	$3900\;rad/s^{2}<\ddot{a}<4500\;rad/s^{2}$
AIS5	$4500\;rad/s^{2}<\ddot{a}$

$\;\ddot{a}$: the head angular acceleration.
